# The Genetic Code Kit: An Open-Source Cell-Free Platform for Biochemical and Biotechnology Education

**DOI:** 10.3389/fbioe.2020.00941

**Published:** 2020-08-19

**Authors:** Layne C. Williams, Nicole E. Gregorio, Byungcheol So, Wesley Y. Kao, Alan L. Kiste, Pratish A. Patel, Katharine R. Watts, Javin P. Oza

**Affiliations:** ^1^Department of Chemistry & Biochemistry, California Polytechnic State University, San Luis Obispo, CA, United States; ^2^Center for Applications in Biotechnology, California Polytechnic State University, San Luis Obispo, CA, United States; ^3^Department of Finance, Orfalea College of Business, California Polytechnic State University, San Luis Obispo, CA, United States

**Keywords:** biochemical education, learn by doing, cell-free protein synthesis (CFPS), *in vitro* transcription and translation, synthetic biology (synbio), central dogma of molecular biology (CDMB), chemical education and teaching, augmented reality (AR)

## Abstract

Teaching the processes of transcription and translation is challenging due to the intangibility of these concepts and a lack of instructional, laboratory-based, active learning modules. Harnessing the genetic code *in vitro* with cell-free protein synthesis (CFPS) provides an open platform that allows for the direct manipulation of reaction conditions and biological machinery to enable inquiry-based learning. Here, we report our efforts to transform the research-based CFPS biotechnology into a hands-on module called the “Genetic Code Kit” for implementation into teaching laboratories. The Genetic Code Kit includes all reagents necessary for CFPS, as well as a laboratory manual, student worksheet, and augmented reality activity. This module allows students to actively explore transcription and translation while gaining exposure to an emerging research technology. In our testing of this module, undergraduate students who used the Genetic Code Kit in a teaching laboratory showed significant score increases on transcription and translation questions in a post-lab questionnaire compared with students who did not participate in the activity. Students also demonstrated an increase in self-reported confidence in laboratory methods and comfort with CFPS, indicating that this module helps prepare students for careers in laboratory research. Importantly, the Genetic Code Kit can accommodate a variety of learning objectives beyond transcription and translation and enables hypothesis-driven science. This opens the possibility of developing Course-Based Undergraduate Research Experiences (CUREs) based on the Genetic Code Kit, as well as supporting next-generation science standards in 8–12th grade science courses.

## Introduction

**FIGURE 1:**
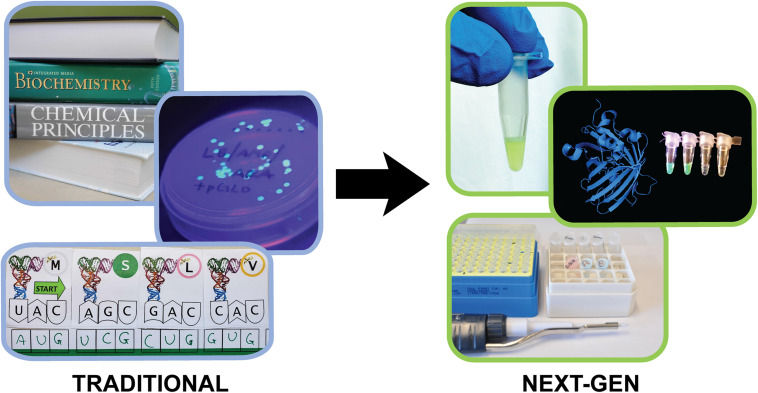
Traditional central dogma teaching tools and the next-generation Genetic Code Kit. The Genetic Code Kit utilizes cell-free protein synthesis and augmented reality to teach the processes of transcription and translation.

Transcription and translation are fundamental cellular processes typically taught in high school and undergraduate science courses and utilized extensively in research settings. As such, students are expected to have an intimate grasp of these concepts to support both their academic and career goals. However, there is evidence that misconceptions about transcription and translation often persist for students even after they have completed these courses (Wright et al., 2014; Newman et al., 2016; Queloz et al., 2017). This issue likely stems from the intangibility of the microscopic processes of the “central dogma” when taught through lecture alone. In the absence of active learning modules, students are unable to visualize and represent these processes for further learning (Kozma et al., 2000; Duncan and Reiser, 2007). To address these limitations and allow students to interact with the individual steps of transcription and translation in the classroom, a variety of model-, analogy-, and virtual- based simulations have been developed (Pigage, 1991; Rotbain et al., 2008; Altiparmak and Nakiboglu Tezer, 2009; Debruyn, 2012; Takemura and Kurabayashi, 2014; Marshall, 2017; Dorrell and Lineback, 2019; Ibarra-Herrera et al., 2019; Chang et al., 2020) (Figure 1). Efforts to develop such activities represent educators’ broad interest in providing students with active-learning modules to improve student learning outcomes. However, chemistry and biology curricula generally rely on laboratory practicals for active learning, as they help students connect scientific concepts and practices. Unfortunately, current wet-lab procedures for teaching transcription and translation are based on bacterial expression of fluorescent proteins, which precludes students from directly accessing and manipulating the genetic code machinery (Ward et al., 2000; Bassiri, 2011; Newman and Wright, 2013; Deutch, 2019) (Figure 1). While all these existing activities are generally low-cost and useful learning tools to help students understand the broad scope and details of transcription and translation, no single activity enables in-depth, hands-on, inquiry-based laboratory learning. The limitations of existing approaches underscore the need for an active learning laboratory-based module that allows students to interrogate transcription and translation in a learn-by-doing fashion.

Active learning has been demonstrated to increase student test scores and decrease the odds of failing classes in science, technology, engineering, and mathematics (STEM) (Nogaj, 2013; Freeman et al., 2014). In addition to these learning benefits, active learning is more engaging for students, ultimately promoting positive attitudes toward their education (Armbruster et al., 2009). Prior work also suggests that active learning may engage underrepresented students more than lecture-based courses, helping to narrow the achievement gap in STEM courses (Haak et al., 2011; Theobald et al., 2020). Curriculum design at our own university has led to the development of studio classrooms for general chemistry courses, which integrate the laboratory and lecture portions of the course into one space and time period. The studio classroom helps students to explicitly connect concepts taught in lecture through experimentation, resulting in improved exam scores, more expert-like learning attitudes, and positive assessments of the active learning environment from both students and instructors (Kiste et al., 2017). In order to apply these findings and address the lack of active learning opportunities for transcription and translation in our biochemistry curriculum, we sought to incorporate cell-free protein synthesis (CFPS) into our classroom laboratories. Toward this end, we developed the “Genetic Code Kit,” a classroom-ready, modular CFPS kit that is amenable to broad dissemination. Importantly, we sought to determine whether implementing the Genetic Code Kit improves student performance on content-based assessments, as well as students’ self-assessed comfort and confidence with experimental procedures.

Advancements in the CFPS platform over the last few decades have enabled a multitude of novel applications in biotechnology, including rapid prototyping for engineering biological systems and easy-to-use point of care diagnostics and biosensors (Pardee et al., 2016; Salehi et al., 2017; Benítez-Mateos et al., 2018; Bundy et al., 2018; Dopp and Reuel, 2018; Takahashi et al., 2018; Gräwe et al., 2019; Gregorio et al., 2019; Silverman et al., 2019; Jung et al., 2020). CFPS generally relies on a cell-extract containing the cellular machinery that supports transcription and translation *in vitro* and is supplemented with additional reagents that provide the necessary energy and precursors. The open nature of the CFPS system is one of the main advantages of the platform as it allows the user to produce proteins on-demand without relying on living cells. Thus, CFPS permits the user to directly manipulate the environment of protein synthesis to suit their needs without the limitation of cellular viability constraints, as is the case for *in vivo* protein expression. The unique advantages of CFPS are also what makes it well suited for active, inquiry-based learning in ways that can transform biochemical and biotechnology education, while simultaneously exposing students to experimental procedures associated with an emerging biotechnology. The pioneering work by BioBits and myTXTL have provided the proof-of-concept in adapting CFPS to classroom settings and engaging students at various grade levels (Huang et al., 2018; Stark et al., 2018, 2019; Collias et al., 2019). Additionally, CFPS remains robust in a variety of chemical environments (Yin and Swartz, 2004; Seki et al., 2008; Dopp et al., 2019; Gregorio et al., 2019; Karim et al., 2020) providing extensive flexibility in accommodating a broad range of learning objectives. These advantages make CFPS a next-generation educational technology to help meet the next-generation science standards. Moving beyond the proof-of-concept, we focus on using CFPS to teach the fundamental processes of transcription and translation and assess the extent and context of learning gains at the undergraduate level.

Transitioning the CFPS platform from a research-focused technology to one that is broadly accessible to high school and university classrooms required extensive simplification, reduced costs, and improved reagent stabilization. Our work to date has taken incremental steps toward these milestones by reducing the number of pipetting steps in CFPS setup (Levine et al., 2019a), creating a less-labor intensive cell extract preparation workflow (Levine et al., 2019b), and identifying a low-cost formulation of additives that enables storage and transport of cell-free extract at room temperature (Gregorio et al., 2020). These advances are part of a concerted effort by the research community to make CFPS accessible to classrooms around the world (Huang et al., 2018; Stark et al., 2018, 2019; Collias et al., 2019). As a result, instructors and institutions now have many options for obtaining CFPS resources for implementation in their classrooms. Each option has its respective advantages that allow instructors to support their learning objectives. Given these combined advancements in accessibility, CFPS is becoming even easier to broadly implement in the teaching laboratory, with the potential for supporting 100s to 1000s of students per quarter.

Here, we report the Genetic Code Kit, an implementation of CFPS used to teach transcription and translation. This kit is intended to be low-cost and open source to support accessibility and broad dissemination, especially to schools and programs with limited funding. To accommodate a variety of curricular limitations, the Genetic Code Kit can be completed within a single 3-h laboratory period, and does not require instructors to dedicate time in a subsequent day to collect data. The kit utilizes crude, *E. coli*-based extract and a DNA template encoding sfGFP, which together have been broadly demonstrated to support robust and reliable protein expression (Park et al., 2017; Levine et al., 2019a,b). Importantly, the sfGFP product resulting from a successful CFPS reaction is easy to visualize in real-time with minimal equipment or processing, and introduces students to a workhorse reporter broadly used in research and industry. The Genetic Code Kit contains 4 components: (1) a tube containing cell extract in which the reaction mixture is to be assembled, (2) the sfGFP DNA template, (3) “solution A” containing cofactors and substrates, and (4) “solution B” containing the energy system. The liquid transfer of just three reagents ranging from 4.2 to 11.4 μL allows students to gain micro-pipetting experience while reducing the likelihood of failed reactions. In our implementation, this setup proved reliable and forgiving, with all students able to obtain visible titers of sfGFP within 90 min. Requiring students to manually add all reagents necessary for transcription and translation is an important aspect of the Genetic Code Kit, as it provides the opportunity to identify and discuss the importance of each class of reagent (e.g., DNA template, energy reagents, and building blocks). This aspect of the kit also provides the flexibility to modify the kit based on the desired learning objectives, allowing for other inquiry-based learning opportunities, as well as CUREs.

We have also developed laboratory materials to accompany the Genetic Code Kit, which help students connect the microscopic processes taking place inside their CFPS reactions to the macroscopic outcome. This includes the laboratory manual and student worksheet (Supplementary Data Sheets 1, 2). Additionally, we created an augmented reality activity that allows students to interrogate the structure-function relationships of GFP to understand the basis for green fluorescence as a function of protein synthesis in their tubes (Supplementary Data Sheet 3). In addition to these specific pedagogical goals related to the central dogma, students also gain exposure to research techniques such as pipetting, reagent handling, the importance of negative and positive controls in experimental design, reaction setup, and data analysis. Importantly, we conducted a controlled study to investigate improvements in student understanding of transcription and translation and their self-assessed comfort with performing an emergent research technique as a function of their hands-on experience with the Genetic Code Kit. Our work demonstrates that implementing CFPS as a hands-on laboratory module leads to significant learning gains associated with transcription and translation learning objectives, as well as positive self-assessment of comfort and confidence with research techniques.

## Materials and Methods

### Cell Growth and Extract Preparation

*Escherichia coli* cell extract was generated using our previously reported CFAI workflow (Levine et al., 2019b). A culture was prepared by inoculating a loopful of BL21^∗^ DE3 cells into a 2 L baffled flask containing 1 L of Cell-free Autoinduction media (5.0 g of sodium chloride, 20.0 g of tryptone, 5.0 g of yeast, 14.0 g of potassium phosphate dibasic, 6.0 g of potassium phosphate monobasic, 6.0 mL of glycerol, 4.0 g of D-lactose, 0.5 g of D-glucose, and nanopure water to 1.0 L). The culture was incubated at 30°C and 200 rpm for approximately 15 h. Subsequently, the culture was centrifuged at 4°C and 5,000 *g* for 10 min. Harvested cells were resuspended in 30 mL of S30 buffer [10 mM Tris OAc, pH 8.2, 14 mM Mg(OAc)_2_, 60 mM KOAc, 2 mM DTT] by vortexing, then spun down at 4°C and 5,000 *g* for 10 min. Supernatant was removed and cell pellets were flash frozen and stored at −80°C or used immediately for extract preparation. Cell pellets were resuspended in 1 mL of S30 buffer per 1 g of cells. 1.4 mL of resuspended cells were aliquoted into a 1.5 mL microfuge tube. The resuspension was sonicated using a Qsonica Q125 Sonicator with a 3.175 mm probe, with the cell resuspension surrounded by an ice water bath. Three pulses of 45 s on and 59 s off, at 50% amplitude were carried out. Immediately after sonication, 4.5 μL of 1.0 M DTT was spiked into the lysate and the tube was inverted several times to mix. Lysate was centrifuged at 4°C and 18,000 *g* for 10 min. The resulting supernatant is the cell extract. The mixture was flash frozen in liquid nitrogen and stored at −80°C until Genetic Code Kit preparation.

### DNA Purification

DNA template pJL1-sfGFP was purified from DH5α cells using an Invitrogen PureLink HiPure Plasmid Maxiprep Kit. DNA was eluted using warm molecular biology grade water instead of the provided TE buffer for compatibility with the CFPS system. DNA plasmid was diluted with molecular biology grade water to a concentration of 42.1 ng/μL, such that no additional water was needed to prepare 30 μL CFPS reactions with a final DNA concentration of 16 ng/μL. DNA was stored at −20°C until Genetic Code Kit preparation.

### Solution A and B Preparation

Solution A (containing cofactors and substrates) was prepared with the following specified concentrations of reagents: 8.14 mM ATP, 5.77 mM GTP, 5.77 mM UTP, 5.77 mM CTP, 153.8 mg/mL folinic acid, 771.9 mg/mL tRNA, 2.71 mM NAD, 1.81 mM CoA, 27.1 mM oxalic acid, 6.79 mM putrescine, 10.2 mM spermidine, 386.9 mM HEPES buffer. Solution B (containing the energy system) was prepared with the following specified concentrations of reagents: 71.6 mM magnesium glutamate, 71.6 mM ammonium glutamate, 930.8 mM potassium glutamate, 14.3 mM 20 amino acids, and 238.1 mM phosphoenolpyruvate. All reagents were dissolved in molecular biology grade water. Both solutions were stored at −80°C until Genetic Code Kit preparation, however, these solutions are also stable at −20°C for 3 months (Supplementary Figure 1).

### Genetic Code Kit Preparation and Reaction Setup

Each kit contained the appropriate amount of pre-aliquoted reagents for the laboratory size and was stored at −20°C for up to 5 days until student use. Each pair of students was provided a strip of four PCR tubes, each containing 10 μL of extract. Each group of four students shared a set of PCR tubes containing molecular biology grade water, pJL1-sfGFP DNA plasmid, solution A, and solution B. Students added 11.4 μL of water, 4.4 μL of solution A, and 4.2 μL of solution B to two tubes as negative controls and 11.4 μL of DNA plasmid, 4.4 μL of solution A, and 4.2 μL of solution B to two tubes as positive controls. All reagents were kept on ice throughout reaction setup. The completed reactions were placed in a 37°C incubator and checked intermittently for green fluorescence. Necessary equipment includes a p20 pipette, pipette tips, an incubator, and a blue or black light. More details can be found in the laboratory manual (Supplementary Data Sheet 1).

### Development of Lab Materials

The lab manual and worksheet (Supplementary Data Sheets 1, 2) for the Genetic Code Kit were developed with the following student learning objectives as a framework: (A) illustrate and describe the processes of transcription and translation; (B) identify the minimally necessary genetic components, enzymes, and reagents necessary for transcription and translation *in vitro*; (C) predict and visualize the outcomes of adding, or not adding, various components to CFPS reactions; (D) define CFPS and its advantages over *in vivo* protein synthesis; (E) paraphrase how energy metabolism sustains transcription and translation in a CFPS reaction. Background on CFPS, the processes of transcription and translation, including the necessary components for each of these processes, and the energy metabolism system operating in CFPS reactions was provided in the lab manual (Supplementary Data Sheet 1).

The student worksheet contained open-ended questions corresponding to each of the learning objectives; some questions also required students to draw a schematic to represent their understanding of a topic (Supplementary Data Sheet 2). For example, for learning objective B, students were asked to illustrate the templates for transcription and translation, including genetic elements like a promoter and ribosomal binding site, and their relative locations to one another on a DNA template. Students were asked to consider the outcome of the experiment if certain elements were missing, such as dNTPs or a particular amino acid, in order to address learning objective C. Questions related to learning objective E focused on steps that require energy input, and how the levels of high-energy molecules like ATP change throughout the CFPS reaction.

The student questionnaire contained 16 content-based questions and 12 attitudinal questions (Supplementary Data Sheet 4). All questions were multiple choice. The content-based section contained three baseline questions that tested knowledge independent of the intervention’s learning objectives and were not expected to be impacted by this laboratory exercise. They acted as a control for differences in baseline aptitudes between the pre- and post- questionnaires. Of the remaining 13 content-based questions, four questions tested transcription knowledge and nine tested translation knowledge. Transcription questions focused on key enzymes and required genetic elements on the DNA template for initiation and termination of transcription. Translation questions were focused on the basic mechanism of the ribosome, including how tRNA and mRNA interact, and the required genetic elements on the mRNA template for initiation and termination of translation. The 12 attitudinal questions asked students to rank their knowledge of transcription and translation vocabulary and comfort with research techniques.

The augmented reality activity utilized Augment^1^, a smart phone application, to project the three-dimensional structure of sfGFP onto student benchtops for an exploration of protein structure, structure-function relationships, and the structural basis for fluorescence (Supplementary Data Sheet 3). However, our pre- and post- questionnaire did not assess student understanding of sfGFP structure or structure-function relationships, so the impacts of this activity on student learning cannot be reported here.

### Implementation of the Genetic Code Kit and Data Collection

The Genetic Code Kit and relevant assessments were implemented in the laboratory component of our non-majors’ “Survey of Biochemistry and Biotechnology” course (CHEM 313) taught by biochemistry faculty. The prerequisite for enrollment was the completion of an introductory organic chemistry course. Our curriculum allows students to select either Organic Chemistry I (CHEM 216), which is the first quarter of a year-long organic chemistry sequence or Survey of Organic Chemistry (CHEM 312), which is a one-quarter survey of organic chemistry (Table 1). The students involved in this study represent a breadth of educational backgrounds, with diverse majors from four colleges at Cal Poly SLO (Table 1). All student data was used with written consent of the participants in the study, based on Institutional Review Board (IRB) approval obtained prior to execution.

**TABLE 1:** Student population distributions by major and completed courses.

**College**	**Major**	**Control**	**Intervention**	**Ochem I**	**Survey of Ochem**
College of Science and Mathematics	Biological Sciences	6	16	17	7
	Kinesiology	0	1	0	1
	Marine Science	0	1	1	0
	Microbiology	1	3	4	0
College of Agriculture, Food, and Environmental Science	Animal Science	2	7	2	7
	Food Science	0	3	0	3
	Nutrition	2	14	2	13
	Wine and Viticulture	2	5	0	6
College of Engineering	Biomedical Engineering	1	2	3	0
	Materials Engineering*	0	1	1	1
College of Liberal Arts	Psychology	1	1	1	1
	Total students	15	54	31	39

*The materials engineering student took both Ochem I and Survey of Ochem.

Implementation occurred over a 3-week period, with each lab section meeting once a week for 3 h. As a “pre-questionnaire” in week 1, all students completed the questionnaire described above (Supplementary Data Sheet 4). In week 2, students in the intervention group used the Genetic Code Kit in their regularly scheduled lab section (Supplementary Data Sheets 1–3). The control group did not meet and did not perform the experiment or augmented reality activity due to a holiday. However, they were provided with the lab manual and completed the same post-lab worksheet. In week 3, all students repeated the same questionnaire administered in week 1, representing the “post-questionnaire.” A total of 69 students completed both pre- and post- laboratory questionnaires, with 15 in the control group and 54 in the intervention group.

Intervention group students performed the Genetic Code Kit lab module in a single 3 h lab period. They were provided the lab manual at least 3 days prior to performing the experiment. After a brief introduction to the experiment in the lab period, students were asked to follow the instructions for reaction setup described in the lab manual, commencing *in vitro* transcription and translation. Reaction tubes were then placed in a 37°C mini-incubator for 1–1.5 h (Supplementary Figure 2). During the incubation period, students completed the post-lab worksheet and augmented reality activity (Supplementary Data Sheets 2, 3) and listened to a short lecture from instructors on the basics of transcription and translation. This brief lecture reviewed information on transcription and translation that was also covered in the 4-h per week lecture portion of the course, and introduced the components of each of the solutions in the Genetic Code Kit that correspond to these processes. This information was also available to students in the control group in the form of the introduction in the lab manual, and in the course textbook. At the end of the incubation period, students visualized fluorescence with the naked eye, and enhanced visibility was achieved using a handheld blue light before the lab period was over.

### Statistical Methods

Student responses were collected and all anonymized assessment scores and responses can be found in Supplementary Table 1. Content-based questions were divided into baseline (1, 5, 6) and transcription and translation (2–4, 7–16) categories based on each question topic. Each category was analyzed by comparing the pre- and post- questionnaire scores for the control and intervention groups, visualized via box and whisker plots generated in SigmaPlot. Paired *t*-tests were run for both groups using JMP, and *p*-values were recorded with a significance level of 0.05. These categories were also analyzed by calculating the normalized learning gain and effect size for both student groups to understand the magnitude of the effect of the Genetic Code Kit. Normalized gain enables the comparison of groups that start at different levels of performance, as it calculates the score increases with respect to the window of potential learning based on pre-questionnaire scores (Hake, 1998). Effect size provides an additional metric that accounts for the number of students tested and the variation in scores among the students (Cohen, 1988). Question-based normalized gain was calculated to determine student performance on each of the 16 questions individually. This metric uses the same equation as normalized gain, however, the average pre- and post- scores are replaced by the percentage of students who answered the question correctly on the pre- and post- questionnaires. Additionally, the content-based data was matched to student major and previous course completion data in the form of an Excel dashboard that allows the user to analyze trends that occur within these subgroups (Supplementary Table 2). The dashboard also allows for a statistical comparison of the control group relative to the intervention group. Due to the different sample sizes, the comparison was performed using the Fisher’s *Z* Test. Point biserial analysis was performed using the Akindi software^2^.

Attitudinal questions were analyzed by comparing the trends in the percentage of students that selected each answer choice on the pre- and post- questionnaires. For statistical analysis, student answers were converted to numerical values, where *A* = 1 and *E* = 5. Paired *t*-tests comparing pre- and post- scores for each question were run using JMP and *p*-values were recorded with a significance level of 0.05.

## Results

### Content-Based Assessment of Student Learning

**FIGURE 2:**
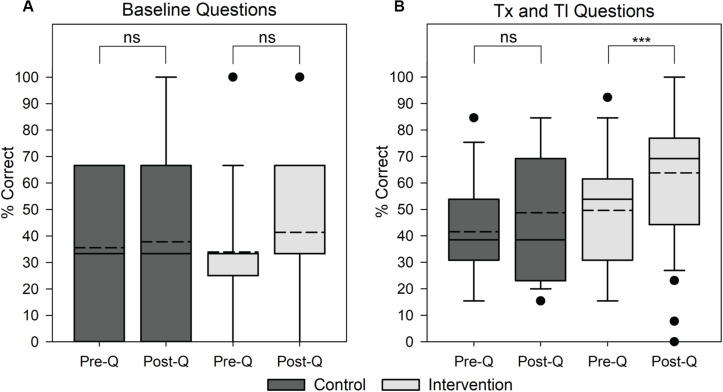
Impact of the Genetic Code Kit on student performance on content-based questions involving baseline or transcription (Tx) and translation (Tl) questions. Student score distributions are depicted as follow: solid lines indicate median, dotted lines indicate mean, boxes demarcate the 25th and 75th percentiles, whiskers represent the 10th and 90th percentiles, and points represent outliers. Control group scores represent a population of 15 students and intervention group scores represent a population of 54 students. The content-based portion of the questionnaire contained 16 questions, 3 baseline and 13 transcription and translation. Specific questions can be found in Supplementary Data Sheet 4. **(A)** Student score distributions for baseline questions. Pre- and post- scores for the control group and intervention group were compared using a two-sided paired *t*-test (ns indicates *p*-value > 0.05) with a null hypothesis that pre- and post- scores will be equal. **(B)** Student score distributions for transcription and translation questions. Pre- and post- scores for the control group and intervention group were compared using a one-sided paired *t*-test (ns indicates *p*-value > 0.05, *** indicates *p*-value < 0.001) with a null hypothesis that pre- and post- scores will be equal.

The content-based section of the questionnaire contained 16 questions (3 baseline, 13 transcription and translation). For baseline questions unrelated to the learning objectives, there was a minimal increase in the mean percentage of correct answers; the control group’s mean score increased from 35.6 to 37.8% and the intervention group’s mean score increased from 34.0 to 41.3% (Figure 2A). However, a two-sided paired *t*-test showed that neither of these increases were significant (*p*-value > 0.05). Thus, we concluded that neither group became significantly better at answering the post-questionnaire as a result of previous exposure in the pre-questionnaire. On transcription and translation questions for the control group, we observed minimal increases in the mean score, from 41.5 to 48.7%. Comparatively, the intervention group had a larger increase in the average score on transcription and translation questions, from 49.6 to 63.8% (Figure 2B). One-sided paired t-tests within the control and intervention groups comparing pre- and post- student scores indicated no significant increase (*p*-value > 0.5) for the control group and a significant increase (*p*-value < 0.001) for the intervention group. This indicates that completing the hands-on Genetic Code Kit experiment significantly improves students’ ability to correctly answer questions regarding transcription and translation.

**FIGURE 3:**
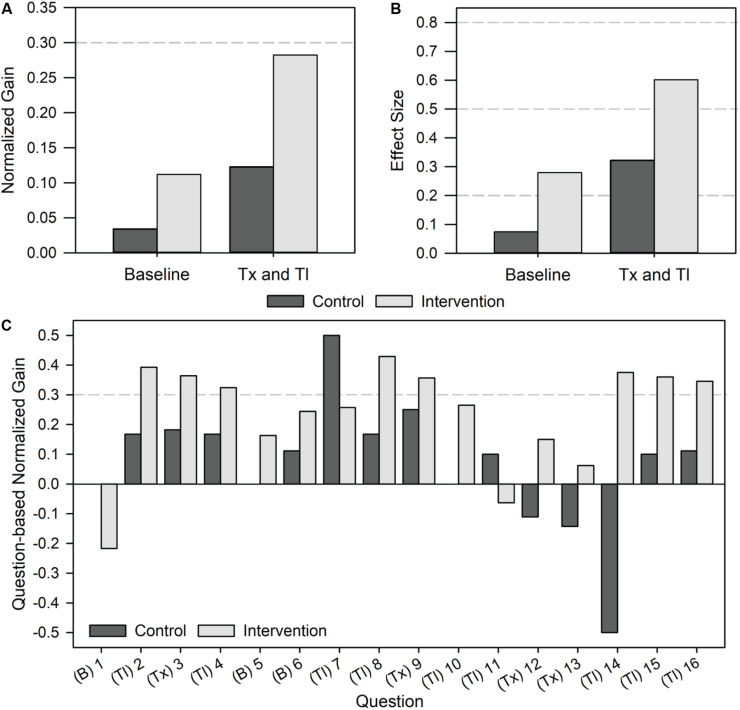
Magnitude of student learning gains on content-based questions upon implementing the Genetic Code Kit. The control group represents a population of 15 students and the intervention group represents a population of 54 students. The content-based portion of the questionnaire contained 16 questions, 3 baseline and 13 transcription and translation. Specific questions can be found in Supplementary Data Sheet 4. **(A)** Normalized gain by question category. Normalized gains > 0.3 indicate a medium gain activity. **(B)** Effect size by question category. Effect sizes of 0.2 indicate small effects, 0.5 indicate medium effects, and 0.8 indicate large effects. **(C)** Question-based normalized gain for each question. Question categories are indicated as follows: (B) baseline, (Tx) transcription, and (Tl) translation. Normalized gains > 0.3 indicate a medium gain activity.

In addition to observing improvements in average assessment scores, we also wanted to better understand the magnitude of the effect of the intervention on student learning gains. Toward this goal, we evaluated both normalized learning gains and effect sizes, since both are commonly used metrics in STEM education. The extent of normalized learning gains is categorized as low (gain < 0.3), medium (0.7 > gain ≥ 0.3), and high (gain ≥ 0.7) (Hake, 1998). On baseline questions, the control and intervention groups demonstrated low gains of 0.03 and 0.11, respectively, as expected (Figure 3A). For the transcription and translation questions, the control group demonstrated a normalized gain of 0.12 while the intervention group demonstrated a gain of 0.28. Effect sizes were also calculated as an additional metric to understand the magnitude of learning gains, while accounting for the student sample size and variation. Effect sizes are categorized as small (effect = 0.2), medium (effect = 0.5), and large (effect = 0.8) (Cohen, 1988). For the baseline questions, we observed small effect sizes of 0.07 for the control group and 0.28 for the intervention group (Figure 3B). Effect sizes on the transcription and translation questions were 0.32 for the control (small-medium) and 0.60 for the intervention (medium-large). As with the normalized gain analysis, the intervention group’s ability to correctly answer questions related to transcription and translation after using the Genetic Code Kit module was much greater than the control group, who did not carry out the activity.

Lastly, we analyzed the question-based normalized gains for each of the 16 questions individually (Figure 3C). This analysis was intended to indicate student performance on individual questions, allowing us to identify questions that were poorly designed or not well-addressed by the Genetic Code Kit. The outcome of question-based normalized gain assessment was the identification of questions 7 and 11 as particularly challenging for the intervention group. In fact, the control group outperformed the intervention group on those two questions, and the normalized gain for the intervention group was negative for question 11. Quantitatively, the point-biserial correlation coefficient values for questions 7 and 11 were above 0.2, suggesting that they are “fair” questions. Qualitatively, it is possible that these questions were written ineffectively, were mismatched with our learning objectives, or that CFPS was not able to resolve student misconceptions regarding the macromolecular interactions involved in translation. In fact, non-covalent interactions involved in translation were not explicitly covered in the pre-lab lecture, worksheet, or lab manual.

Given that we observed meaningful normalized learning gains and effect sizes upon intervention despite questions 7 and 11, we remained curious about the learning gains observed in the remaining questions. In a follow-up analysis (Supplementary Tables 2, 3), we removed questions 7 and 11 from the group of transcription and translation questions and used this narrower scope to evaluate learning gains by student demographics. We observed that students who had previously taken Ochem I, the first quarter in a year-long series of organic chemistry, had significantly higher learning gains compared to the control group (*p*-value < 0.05), while those who had taken Survey of Ochem did not significantly benefit (*p*-value > 0.05) from the Genetic Code Kit intervention compared to the control group (Supplementary Table 3). The intervention group students that did not significantly benefit were mostly from the College of Agriculture, Food, and Environmental Science, who have historically underperformed in the Survey of Biochemistry and Biotechnology course. While this observation is only suggestive when we removed questions 7 and 11 from the analysis, it represents an intriguing starting point for using CFPS to consider preparation gaps and achievement gaps within our student populations. These results suggest that if question design can be improved and sample size can be increased, implementation of CFPS has the potential to explore the basis for preparation and achievement gaps in biochemical education. Regardless, these additional findings are contingent on solving the learning issues identified in questions 7 and 11, as these differences in prerequisite preparation only appear when they are removed from the analysis.

Overall, significant increases in the average scores on content-based questions (Figure 2), a normalized learning gain around 0.3, and an effect size of 0.6 for the intervention group (Figure 3) indicate that implementing the Genetic Code Kit improved students’ ability to comprehend and answer questions relating to transcription and translation. As no significant increase (*p*-value > 0.05) in the performance on baseline questions was observed, we propose that the observed increase in assessment scores for transcription and translation questions was a result of the Genetic Code Kit rather than repeated exposure to the questionnaire.

### Attitudinal-Based Assessment of Student Learning

The pre- and post- questionnaires completed by both the control and intervention groups contained a total of 12 attitudinal questions. These questions prompted students to self-assess their recognition and knowledge of transcription and translation vocabulary, as well as their comfort with laboratory techniques used in CFPS. Prior work has documented students’ deficiency in metacognitive skills and found that active learning pedagogies can strengthen these skills (National Research Council, 2000). Our attitudinal-based questions allow us to examine how students’ perceptions of their learning correlate with their results on the content-based assessment (Supplementary Figure 3). We found that both the control and intervention groups showed positive correlations on pre- and post- questionnaires, with an increase in the slope from pre- to post- questionnaire. For the control group, the pre-questionnaire *R*^2^-value was 0.02 and post-questionnaire was 0.36. For the intervention, the pre-questionnaire *R*^2^-value was 0.10 and post-questionnaire was 0.30. The relatively low pre-questionnaire *R*^2^ is noteworthy: it shows that students’ knowledge and attitudes are, effectively, uncorrelated. The increase in post-questionnaire *R*^2^ indicates that knowledge and attitudes move in the same direction. Overall, this analysis indicates that students’ self-reported confidence correlated with their performance on content-based questions. As a result, we pursued more detailed analysis of the attitudinal-based questions.

**FIGURE 4:**
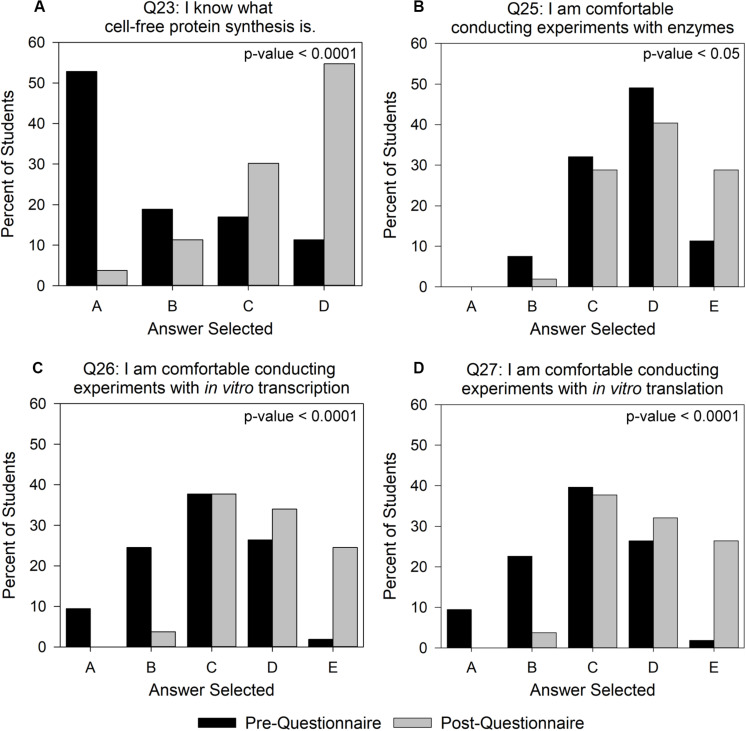
Changes in intervention group student attitudes toward CFPS and conducting CFPS-based experiments. Answer choices for **(A)** ranged from A – “I have no idea what this term means” to D – “I know what this term means.” Answer choices for **(B–D)** ranged from A – “Strongly disagree” to E – “Strongly agree.” Student answers were converted to a numerical value where *A* = 1 and *E* = 5, in order to calculate p-values using a one-sided paired *t*-test with a null hypothesis that pre- and post- scores would be equal. The intervention group contained 52 students. This is less than the number of students in the content analysis, as some students did not complete the attitudinal section of the post-questionnaire. All possible answer categories can be found in Supplementary Data Sheet 4.

We first considered the possibility that improvements in students’ self-assessment of their confidence were an outcome of their recognition of vocabulary terms through repeated exposure to the questionnaire rather than as a result of improved conceptual understanding of the terms. To address this concern, we chose to perform detailed per-question analysis for the attitudinal-based assessment on questions that involved comfort with CFPS as an indicator of how beneficial the activity was in introducing a novel biotechnology. For the intervention group, we observed significant increases (*p*-value < 0.05) between pre- and post- scores for questions 23 and 25–27 using a one-sided paired *t*-test (Figure 4). When prompted with “I know what CFPS is” (question 23) on the pre-questionnaire, over 50% of the intervention group students indicated that they had no idea what the term meant and ∼11% indicated that they knew what the term meant (Figure 4A). After conducting the experiment, this changed to less than 5% and greater than 50%, respectively. The control group saw a similar, but less extensive shift in the trend with almost 40% of students reporting that they knew what the term meant in the post-questionnaire (Supplementary Figure 4). The comparable shift in the control and intervention groups is likely due to the background information that they received on CFPS through the lab manual alone. For question 25 (Figure 4B), “I am comfortable conducting experiments with enzymes,” pre-questionnaire comfort was generally high for the intervention group, but only ∼11% of students indicated that they “strongly agreed.” However, the Genetic Code Kit increased intervention student confidence in working with enzymes, such that almost 30% of students said they “strongly agreed” on the post-questionnaire. This was a noteworthy observation, since the Genetic Code Kit was implemented at the end of the quarter, and students had worked with enzymes in numerous previous laboratory modules. The intervention group’s comfort with conducting experiments involving *in vitro* transcription and translation (questions 26 and 27; Figures 4C,D) also showed notable improvement, with the number of students answering “strongly agree” increasing to ∼25% from less than 2%. Comparatively, the control group had less than 8% of students say that they “strongly agreed” in response to questions 25–27 (Supplementary Figure 4). These data indicate that the intervention group’s hands-on exposure to the CFPS reaction improved their comfort with these laboratory skills over the control group.

## Discussion

The CFPS platform has seen significant development and widespread use as a biotechnology tool in recent years. CFPS harnesses the genetic code in a test-tube, in a flexible and tunable biochemical milieu, making it poised to be a transformative educational technology. Specifically, CFPS allows students to probe the processes of transcription and translation in a way that improves their learning outcomes, while providing them the technical skills for careers in biotechnology. Here, we report the implementation of our Genetic Code Kit, a simplified, yet modular CFPS reaction, in college-level biochemistry curriculum. Importantly, the Genetic Code Kit improved students’ understanding of transcription and translation for undergraduate students in a survey of biochemistry course. Our results suggest that the tactile process of setting up a CFPS reaction by adding solutions containing the building blocks, energy system, and DNA template to *E. coli* extract, and observing the real-time production of a fluorescent protein increases students’ comprehension of transcription and translation. Our observations are consistent with the extensive literature on the benefits of a physical experience in student learning (Bopegedera, 2011; Zacharia et al., 2012; Kontra et al., 2015; Kiste et al., 2016). Moreover, the Genetic Code Kit may help resolve common student misconceptions surrounding transcription and translation. For example, physically supplementing the CFPS reaction vessel with amino acids may eliminate potential confusion on the source of amino acids or the misconception that translation produces amino acids (Fisher, 1985). Additionally, requiring students to add both DNA and nucleotides to the CFPS reaction vessel could help resolve student misconceptions that DNA is converted into RNA via a chemical reaction instead of being used as a template for a new nucleotide strand (Wright et al., 2014).

The shifts in responses to attitudinal-based questions showcase the usefulness of the Genetic Code Kit to prepare students for future careers in laboratory science. Notably, these benefits to students extend beyond the learning gains in the content-based questions to support increased student confidence with the laboratory techniques used for CFPS. This work suggests that improvements to familiarity with biotechnologies and comfort in implementing biotechnology-based experiments provide fundamental advances toward workforce development. Prior work has documented that exposing students to research as part of science curriculum has improved student engagement in research outside of the classroom (Lindsay and McIntosh, 2000). Furthermore, undergraduate involvement in research experiences is known to increase student interest in obtaining a Ph.D. and pursuing a STEM field, especially when students are invested and interested in their research (Russell et al., 2007).

In order to enable all students to access these learning outcomes, the Genetic Code Kit is designed to be a low-cost, easy to assemble and implement, highly tailorable platform for various curricula and learning objectives, and requires minimal training and equipment. The Genetic Code Kit costs $4.08 per student, based on 4x CFPS reactions per student (Table 2). The cost of $1.02 per 30 μL reaction is inclusive of all materials, reagents, and labor at an estimated rate of $25/hr for the technician’s efforts. The development of the previously reported CFAI workflow has allowed us to significantly reduce the time required for cell extract preparation, reducing the cost associated with labor (Levine et al., 2019b). For example, preparing kits for 375 students requires under 25 person-hours. Notably, our kit preparation can be completed entirely by undergraduate students, as was done in this work, which significantly reduces the cost of implementation. The Genetic Code Kit preparation is also highly scalable. In fact, preparing larger quantities becomes more cost-effective. After the cost of labor, the next largest expense is the energy reagents that drive the PANOxSP-based CFPS reaction, but prior work has shown that this cost could be further reduced by leveraging glucose metabolism (Calhoun and Swartz, 2005). Instructors and institutions now benefit from a variety of CFPS options for their classrooms and Table 2 provides a list of options to choose from. We include cost comparisons in Table 2, since this may be one possible driver for selecting a path to implementing CFPS. However, we urge instructors to review the benefits of all listed options, as they may outweigh costs, particularly for convenience of implementation or suitability to specific learning objectives.

**TABLE 2:** Cell-free protein synthesis (CFPS) reaction costs for in-house and commercially available kits.

**Product**	**Vol/rxn (μL)**	**Cost/rxn**	**Cost/student**	**Cost/100 students**	**References**
Genetic Code Kit	30	$ 1.02	$ 4.08	$ 408	Levine et al., 2019b
miniPCR BioBits	7	$ 2.97	$ 11.88	$ 1,235	Stark et al., 2018
Bioneer AccuRapid Midi	30	$ 2.94	$ 11.76	$ 1,544	–
Promega S30 for Circular DNA	30	$ 9.86	$ 39.44	$ 3,944	–
Arbor myTXTL	12	$ 10.65	$ 42.60	$ 4,260	Collias et al., 2019
NEBExpress	30	$ 10.20	$ 40.80	$ 5,100	–
Thermo Expressway Maxi	25	$ 13.20	$ 52.80	$ 5,280	–
Sigma iPE-Quick Kit	30	$ 12.42	$ 49.68	$ 5,400	–

The Genetic Code Kit can be tailored to meet a variety of learning objectives beyond teaching transcription and translation. The open nature of the system makes it poised to support inquiry-based learning at a variety of grade levels and CUREs through minor modifications to the reaction setup or DNA template described here. These possibilities can help tailor the kit to the desired grade level and course learning objectives, and include (1) the sequence-function relationships of various genetic elements such as promoters, ribosome binding sites, and codon optimization, (2) riboswitches and aptamers, (3) genetic circuits, (4) CRISPR, (5) probing the mechanisms of various antibiotics, such as protein synthesis inhibitors, and many more. Some unique applications of CFPS for classroom instruction have already been developed for the BioBits kits (Huang et al., 2018; Stark et al., 2018, 2019). Lastly, the Genetic Code Kit can be implemented as a free-standing laboratory module to fit within a single 3-h lab course, but it can also be integrated into existing curricula. For example, this lab could be preceded by molecular biology labs including PCR or CRISPR and followed by analysis of the protein product via other traditional biochemical methods such as western blotting, ELISA, or SDS-PAGE.

Overall, this work represents the first controlled study of student learning gains resulting from a hands-on, learn-by-doing intervention based on CFPS. While this study’s findings are limited by a small sample size and focus on undergraduate students from a single institution, we observed significant gains for learning objectives relating to transcription and translation. Thus, the results of this work provide the foundation to expand assessments of learning gains to various educational levels, pursue multi-institutional efforts that include large student sample sizes, and iterate on the design of the kit to further improve student learning gains for a broad range of learning objectives. We propose that the expansion of this work will further validate the important role of CFPS in biochemical education while supporting workforce development for the growing biotechnology industry.

## Data Availability Statement

All datasets generated for this study are included in the article/Supplementary Material.

## Ethics Statement

The studies involving human participants were reviewed and approved by human subjects in Research Institutional Review Board at Cal Poly. The patients/participants provided their written informed consent to participate in this study.

## Author Contributions

LW and NG wrote the manuscript, performed statistical analysis, and generated the figures. LW, NG, BS, and WK prepared all reagents for teaching lab implementation. BS and WK performed reagent storage and time-course testing. AK helped to design the teaching lab implementation and guided the analysis and visualization of assessments of student learning gains. PP helped with statistical analysis of the data. JO conceived the project. KW and JO designed and executed the teaching lab implementation. All authors helped to revise the manuscript and agreed to the accuracy of the work reported.

## Conflict of Interest

The authors declare that the research was conducted in the absence of any commercial or financial relationships that could be construed as a potential conflict of interest.

## Footnotes

1 www.augment.com

2 www.akindi.com

## Supplementary Material

Supplementary Data Sheet 5 contains the main Supplementary Materials documentation. Supplementary Data Sheet 6 contains the information for Supplementary Table 2.

## Abbreviations

CFPS: cell-free protein synthesis
CUREs: course-based undergraduate research experiences
sfGFP: superfolder green fluorescent protein.
