# Supplementary material for: The Genetic Code Kit: An Open-Source Cell-Free Platform for Biochemical and Biotechnology Education
Source: Front Bioeng Biotechnol. 2020 Aug 19;8:941. doi: 10.3389/fbioe.2020.00941 (PMC7466673; doi:10.3389/fbioe.2020.00941)
Supplement: Supplementary file 2 [file Data_Sheet_1.pdf]

Genetic Code Lab

This lab was developed by undergraduate students:
W. Y Kao, N. E. Gregorio, L. C. Williams, and B . So.

Overview

This experiment is intended to allow interrogation of the biochemical processes of transcription and translation, give hands-on experience with an emergent research technology, and provide practice with experimental setup and technique. You will learn about cell-free protein synthesis, including how it works, how to set it up, and why it is important.

Learning Objectives

A. Illustrate and describe the processes of transcription and translation.
B. Identify the minimally necessary genetic components, enzymes, and reagents necessary for transcription and translation in vitro.
C. Predict and visualize the outcomes of adding, or not adding, various components to cell free reactions.
D. Define cell-free protein synthesis and what advantages it has over in vivo protein synthesis.
E. Paraphrase how energy metabolism sustains transcription and translation in a cell free protein synthesis reaction.

Purpose

To produce green fluorescent protein (GFP) using cell-free protein synthesis and visually assess protein production in comparison to a negative control through the appearance of a green color.

Background

<u>Transcription and Translation:</u>

The processes of transcription and translation are fundamental to all organisms on Earth as they convert information stored in DNA into proteins which execute cellular functions. Transcription is the first step of gene expression, in which a particular segment of DNA is copied into single strand messenger RNA (mRNA) by the enzyme RNA polymerase. During transcription, a DNA sequence is read by an RNA polymerase, which produces a complementary, antiparallel RNA strand called a primary transcript.

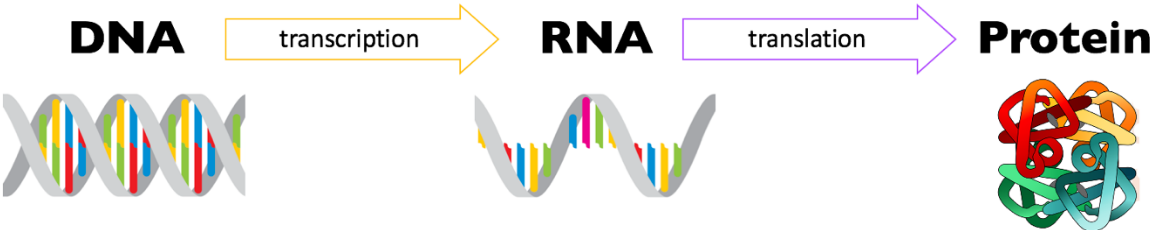

https://www.vecteezy.com/free-vector/dna,
https://commons.wikimedia.org/wiki/File:Main_protein_structure_levels_en.svg

The mRNA then serves as a template for the protein's synthesis through translation. Translation is the process in which ribosomes translate the molecular ‘instructions’ encoded in the nucleic acid ‘language’ into proteins using amino acids building blocks. The polypeptide chain that is produced by the ribosome later folds into protein, which can then properly perform its functions in the cell.

Cell-Free Protein Synthesis:

This genetic code kit leverages Cell-Free Protein Synthesis (CFPS), a research technology that allows proteins to be produced in a test tube, rather than in living cells. This is an important technology because it allows scientists more freedom to manipulate protein expression, in turn allowing for a wider variety of proteins to be studied.

CFPS is achieved by capturing the necessary components for transcription and translation together in a test tube. Many of the necessary components, such as the 20 natural amino acids and RNA nucleotides are readily available for purchase, while cellular machinery like ribosomes are not. Instead, this cellular machinery is obtained by purifying "cell extract" from *E. coli* cells. The cell extract contains the components of a cell that are essential for transcription and translation (such as ribosomes and polymerases), yet no longer contains any of the bacterial cells' genomic DNA or cell walls. The lack of genomic DNA means that cell free protein synthesis can be used to make solely the protein encoded in the DNA template supplied in the reaction. Because the reaction does not rely on the survival of bacterial cells, no other protein synthesis is necessary for the success of the reaction. Moreover, because the cellular machinery is not trapped inside the cell (the cell membrane has been removed), CFPS is an open system that allows for direct manipulation of the environment of protein synthesis. This means that you will be able to control exactly what goes into each reaction, and that CFPS can be tailored to the protein of interest if needed to improve expression.

CFPS is rapidly becoming the standard for making proteins that are difficult to express or are detrimental to a cell *in vivo* because CFPS doesn’t depend on cell survival and can be manipulated in various ways to improve protein synthesis. In *in vivo* systems, the cell is like a black box and the researcher cannot easily manipulate the environment inside of the cell where the protein is produced. *In vivo* expression also relies on growth and division of living cells, so expression of proteins that are burdensome to cells and siphon energy away from the pathways they need to survive, or protein that are toxic to cells cannot easily be expressed *in vivo.* Furthermore, the live cells must be transformed with the plasmid DNA coding for the protein of interest, a step which is not necessary in CFPS. This makes CFPS much faster for expressing a large variety of different proteins and is a key part of the plug-and-play nature of CFPS.

Overall, these advantages are making CFPS a technology that is becoming relevant for personalized medicines, diagnostics, and more. Researchers are using CFPS to more easily produce proteins that were previously difficult or impossible to study using *in vivo* methods, to ensure that protein have the proper post-translational modification for study or to install new modifications using genetic code expansion, to create virus-like particles for improved drug-delivery, to perform high throughput screening of a variety of proteins faster than previously possible, as a scaffold for metabolic engineering, and to create biosensors and point-of-care diagnostics. A few biotech companies, such as Sutro Biopharma and Tierra Biosciences, are using CFPS to create antibody-drug conjugates to fight cancer and identify a new generation of molecules for new applications. There are also CFPS platforms based on a variety of different cell

lines, from bacteria, plants, mammals, and more. CFPS also offers the benefit of hands-on learning of the processes of transcription and translation, as you will experience first-hand in this lab module. The CFPS reaction will allow you to further investigate what is necessary for transcription and translation to take place, as you will have to supplement the cell extract with all the necessary reagents for successful protein synthesis.

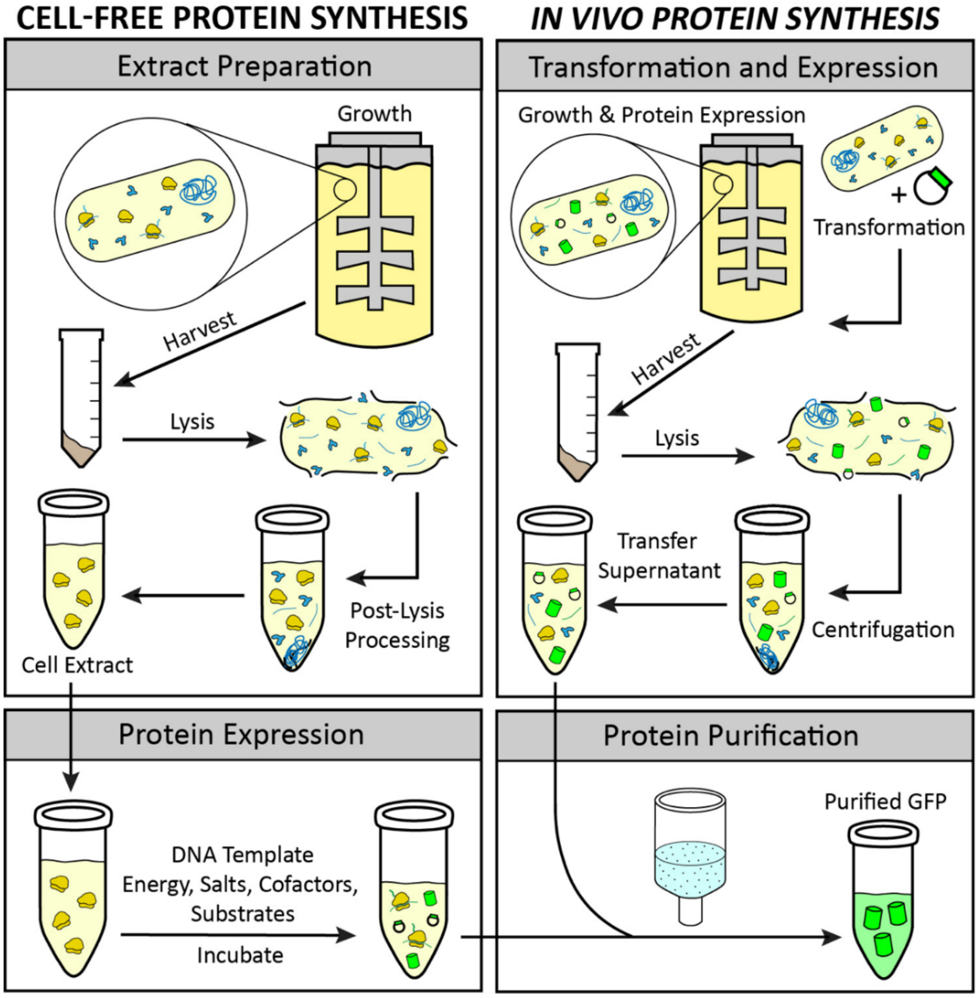

Methods and Protocols, 2(1), 24, doi:10.3390/mps2010024

Reaction Setup:

This schematic outlines the reagents that you will be adding to the cell-free proteins synthesis reaction:

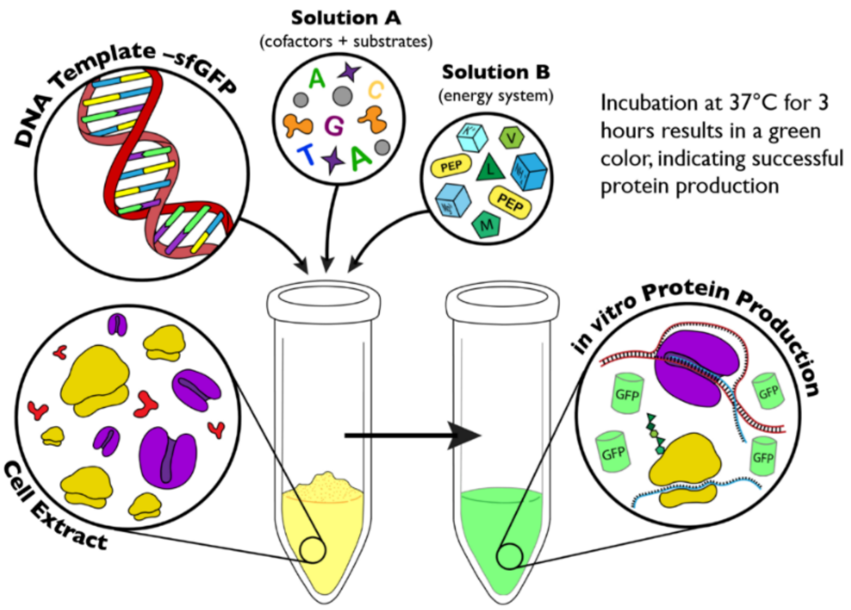

Solution A contains:

- HEPES pH 7.5 Buffer
- 15x master mix – rNTPs (ribonucleotide triphosphates) for mRNA production in transcription, folinic acid, tRNAs for delivery of amino acids to ribosome
- Spermidine - cofactor for transcription
- Putrescine - cofactor for transcription
- NAD+ - coenzyme for metabolism
- Oxalic Acid - energy regeneration
- CoA – coenzyme

Solution B contains:

- Phosphoenolpyruvate (PEP) – the energy source for the reaction
- 20 amino acids – building blocks from which proteins are assembled
- 15x salt solution - potassium glutamate, magnesium glutamate, ammonium glutamate (cofactors)

E. coli Extract contains:

- T7 RNA Polymerase – machinery for transcription
- Ribosomes – machinery for translation
- Various auxiliary proteins that support protein synthesis and energy metabolism (the exact composition is not well characterized)

Cell extract is made by first growing up a culture of *E. coli* cells and washing them with buffer to remove residual media and other metabolic byproducts. Then sonication is used to lyse the cells by disrupting cell membranes using sound waves, which causes the cell to burst. After lysis, the mixture is centrifuged at a high speed for several minutes to pellet out cell membrane, large pieces of genomic DNA, and precipitated proteins. The supernatant becomes the cell extract and is stored at -80 C for future use in CFPS reactions.

The DNA template you will be using is a plasmid containing the gene for Green Fluorescent Protein (GFP), which glows green upon translation. The green fluorescence is visible to the naked eye, and you will use this feature of your protein product to determine that your reaction is successful.

Architecture of *Aequorea victoria* Green Fluorescent Protein

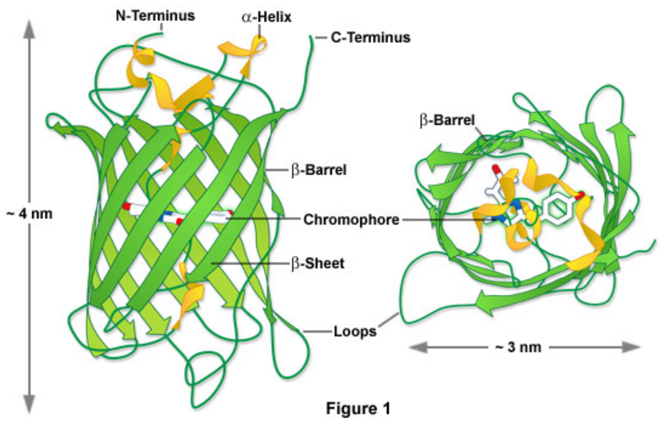

Figure 1

http://zeiss-campus.magnet.fsu.edu/print/probes/jellyfishfps-print.html

Transcription:

Transcription minimally requires a DNA template, ribonucleotide building blocks, RNA polymerase and a buffered solution containing cofactors. RNA polymerase first binds to a promoter sequence that is found upstream of the start codon in the DNA template. Once the RNA polymerase is bound, it proceeds to make an mRNA 'copy' of the DNA sequence on the template strand of DNA. The mechanism of transcription can be viewed in your textbook on page 661 (Figure 36.3) The mRNA copy is synthesized from the DNA template in the 5'-3' direction, meaning that the new ribonucleotide is always added to the 3' end of the growing chain. Each addition of a nucleotide forms a new phosphodiester bond, and pyrophosphate is released as a byproduct. After the primary transcript is formed, the mRNA has a tendency to form intramolecular hydrogen bonds – in other words, it tends to form base pairs with itself. A primary transcript that is folded in this way isn't very useful for translation, thus two molecules are added to the CFPS mixture to stabilize the primary transcript in its unfolded form: putrescine and spermidine. These molecules form hydrogen bonds with the unfolded mRNA, preventing it from forming base pairs with itself.

Transcription proceeds well past the stop codon of a gene (TAG, TAA, TGA). The RNA Polymerase will dissociate from the DNA template when it reaches a sequence known as the terminator. The sequence of the terminator depends on the mechanism of termination, but is always located many base pairs downstream of the stop codon.

Translation:

With the primary transcript in solution, now the system is primed for translation. Translation has more requirements than transcription. Translation requires an mRNA template, amino acid building blocks, the ribosome, and a buffered solution containing cofactors. However, the ribosome requires amino acids to be 'delivered' to the catalytic active site in a different form; they must be covalently bound to transfer RNA (tRNA) to be used in translation. Enzymes called aminoacyl tRNA synthetases (aaRS) are responsible for creating the covalent bond between amino acids and their complementary tRNAs. The *E. coli* cell extract contains tRNA, aaRS's, and amino acids are added to the reaction in Solution B.

The ribosome is composed of 2 separate subunits made up of protein and ribosomal RNA (rRNA). These two subunits assemble on the mRNA template at the ribosomal binding site, also known as the Shine-Dalgarno sequence which is upstream of the AUG start codon in the mRNA sequence. To initiate translation in bacteria such as *E. coli*, the ribosome pairs the AUG start codon with a tRNA carrying a derivative of methionine, known as *N*-formyl methionine. In order to ensure that translation initiation is not stalled in the CFPS reaction, the CFPS reaction is supplemented with the molecule folinic acid (present in Solution A) which is a precursor for *N*-formyl methionine synthesis. Once translation is initiated, the ribosome continues to catalyze peptide bond formation between amino acids linked to their respective tRNA's, which are base paired through hydrogen bonds with the mRNA template. Please review the mechanism for translation on pages 725-727 in your textbook. The ribosome continues to catalyzed translation until a stop codon is reached on the mRNA template (UAG, UAA, UGA).

CFPS Metabolism:

While the cellular machinery needed for transcription and translation itself are essential to successful CFPS reactions, an *in vitro* system for energy production is equally important. The CFPS reactions that you will run today utilizes an energy system called "PANOxSP" (Phosphoenolpyruvate, Amino acids, NAD^+, Oxalic acid, Spermidine, Putrescine). The full list of components needed for the PANOxSP system were outlined above. Overall, these components work together to regenerate the metabolic pathway illustrated below, which allows for formation of ATP *in vitro*.

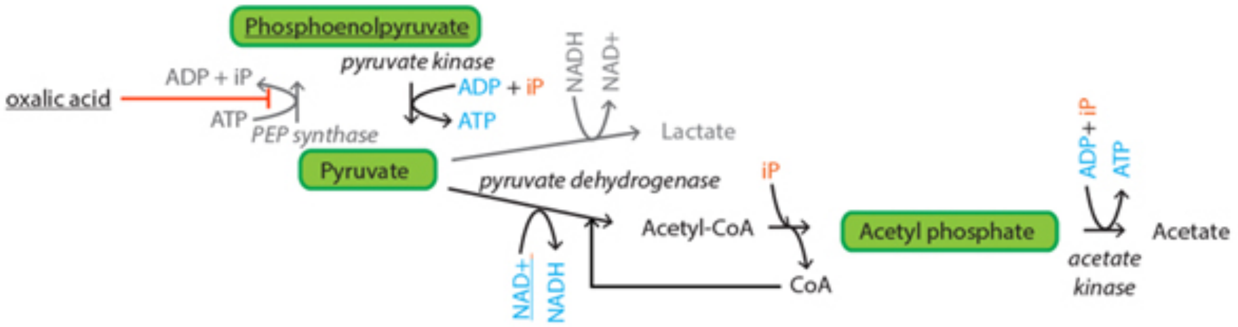

PANOx energy regeneration metabolism. Underlines: PANOx metabolites (amino acids not shown). Italics: enzymes names. ATP is generated from the conversion of PEP to pyruvate by pyruvate kinase, and the subsequent conversation of acetyl phosphate to acetate by acetate kinase. *AIChE Journal, 58(1), 5–13.*

As ATP is consumed during protein synthesis, ADP and inorganic phosphate (Pi) accumulate in the reaction. Once all ATP is consumed, the reaction comes to a stop. Regenerating ATP from ADP and Pi would extend the lifetime of the reaction and result in more protein product. Mimicking the metabolism inside the cell, this is accomplished in two ways. First, phosphoenolpyruvate (PEP) is added to the reaction for substrate-level phosphorylation of ADP to make ATP. The addition of oxalic acid prevents consumption of the ATP produced in this reaction, by inhibiting the reverse reaction. The reaction that forms ATP also forms pyruvate, which can proceed through the bridge step between glycolysis and the Kreb's cycle to form acetyl-CoA. NAD^+ is needed as a cofactor for this transformation. Additional ATP can be generated if the acetyl-CoA is transformed into acetate. Second, some evidence has led researchers to believe that oxidative phosphorylation may also take place in CFPS. Together, the starting concentrations of ATP, substrate level phosphorylation, and ongoing oxidative phosphorylation provides the energy necessary for transcription and translation to take place in a test tube, just as they would inside of our cells.

Procedure

Safety Considerations: The hazards of reagents involved in this experiment are minimal due to such small volumes. However, all solutions should still be handled with care. Additionally, appropriate personal protective equipment (PPE) should be worn, including gloves. Gloves will not only protect you from the reagents, but they will protect the reagents from you! Because CFPS requires properly functioning RNAs, you must avoid introducing any outside RNAses that may prevent the reaction from working, and your hands are covered in RNAses!

Reaction setup:

**Negative**					
Label:	Extract (uL)	H_20 (uL)	DNA (uL)	Solution A (uL)	Solution B (uL)
–	10.0	11.4	0	4.40	4.20
–	10.0	11.4	0	4.40	4.20
**Positive**					
Label:	Extract	H_20 (uL)	DNA (uL)	Solution A (uL)	Solution B (uL)
+	10.0	0	11.4	4.40	4.20
+	10.0	0	11.4	4.40	4.20

1. Obtain four tubes containing 10 uL of extract.
2. Label two tubes with " – " indicating negative controls.
3. Label the remaining two tubes with "+" indicating your positive controls.
4. Add 11.4 uL of H_2O to the two **negative** control tubes. DNA will not be added to the negative control.
5. Add 11.4 ul of **DNA** encoding GFP to the **positive** control tubes.
6. To all tubes, add 4.40 uL of Solution A.
7. To all tubes, add 4.20 uL of Solution B.
8. After all reagents have been added to the tubes, gently tap the tube on the bench top to ensure that all liquid coalesces at the bottom of the tube. This is important because all of the reagents must be present for a successful reaction! Observe that the final volume is approximately equal in all tubes. This is important because it ensures that all of the reagents are present in the correct concentrations.
9. After all droplets are in a single bead at the bottom of the tube, gently flick the tube to thoroughly mix the added reagents together while keeping them in a single bead. Alternatively, you can pipette the reaction contents up and down to mix them.
10. Place the tubes in an incubator at 37°C. for 2 hours.
    a. Alternatively, wrap the tubes in parafilm and then in a clean paper towel and place them in your pocket to incubate.
11. After incubation, you should be able to observe a green color change in your experimental condition, indicating that GFP protein was successfully produced.
