# Supplementary material for: The Genetic Code Kit: An Open-Source Cell-Free Platform for Biochemical and Biotechnology Education
Source: Front Bioeng Biotechnol. 2020 Aug 19;8:941. doi: 10.3389/fbioe.2020.00941 (PMC7466673; doi:10.3389/fbioe.2020.00941)
Supplement: Supplementary file 3 [file Data_Sheet_2.pdf]

Genetic Code – Worksheet Name:________________Section: __________

1. **In this experiment, you were given the** *E. coli* bacterial genetic code in a test tube. Upon adding your DNA template, you initiated the following reactions; DNA -> RNA -> Protein. Illustrate each step of the process as it is occurring in your test tubes. Label all components required for each process including building blocks (NTPs, amino acids, etc), tRNAs, and enzymes (include specific names of enzymes).

DNA -> RNA: Transcription

RNA -> protein: Translation

2. The DNA template used to initiate the transcription reaction is a plasmid, small circular DNA. For the cartoon below representing the circular plasmid DNA containing your gene, draw the additional genetic elements that would be required for transcription to start and stop correctly (assume the gene is read from left to right in the figure). Additionally, describe the role of these genetic elements in transcription.

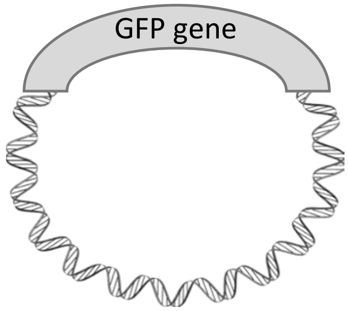

3. Once the gene is successfully transcribed, mRNA is generated. In the mRNA cartoon below, draw the genetic elements that would be required for translation to start and stop correctly. Describe the role of each genetic element and how they function. Again, assume the mRNA is translated left to right in the schematic below.

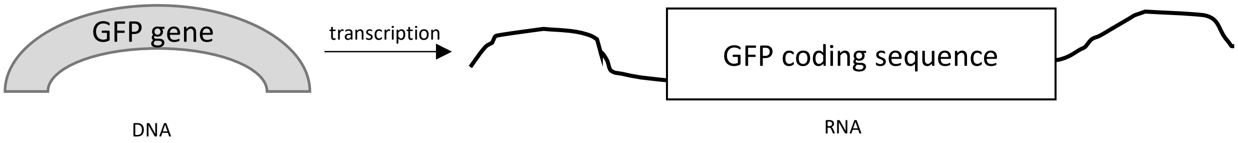

4. The person preparing Solution B for today's lab made an error and forgot to include alanine, how would the outcome of the reaction be affected?

5. The person preparing Solution A for today’s lab made an error and included dideoxyribonucleotide triphosphates (ddNTPs) instead of ribonucleotide triphosphates (rNTPs). See these structures below What effect would this have on the outcome of your experiment?

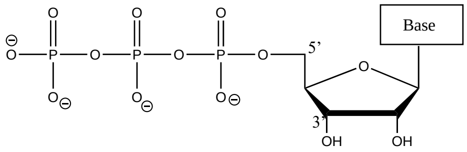

rNTP

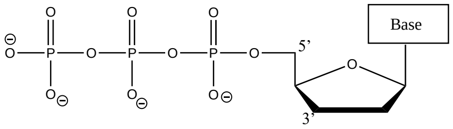

ddNTP

6. HEPES acts as a buffer within the CFPS reaction. What would happen to the components of the reaction if it was not present?

7. CFPS reactions require energy input to proceed through protein synthesis. In your reactions, you added phosphoenolpyruvate (PEP) as the source of energy. Typically, we say the ATP is used as an energy source in the cell. Using the structures below, identify the key properties of PEP that enable it to be a source of energy.

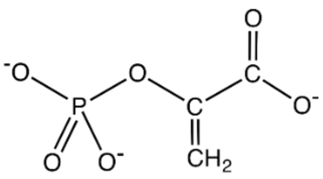

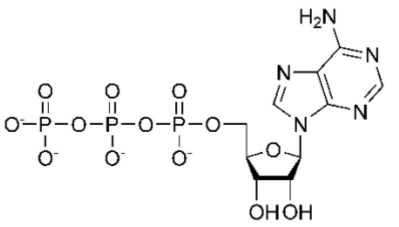

PEP

ATP

8. Identify two steps in the CFPS procedure that require the input of energy in the form of ATP. For each, explain why this step requires an energy input. Be as specific as possible.

9. Based on your answer to questions 2 and 8, explain why ATP levels steadily decrease as the protein synthesis proceeds.

10. What are some possible byproducts that would build up in a cell-free protein synthesis reaction?

11. Were you able to successfully produce GFP protein? How do you know? If you were not able to produce the protein, list some possible reasons why your reaction was not successful.
