# Supplementary material for: The Genetic Code Kit: An Open-Source Cell-Free Platform for Biochemical and Biotechnology Education
Source: Front Bioeng Biotechnol. 2020 Aug 19;8:941. doi: 10.3389/fbioe.2020.00941 (PMC7466673; doi:10.3389/fbioe.2020.00941)
Supplement: Supplementary file 4 [file Data_Sheet_3.pdf]

Green Fluorescent Protein – Augmented Reality Activity

The 2008 Nobel prize in Chemistry was awarded for the discovery and development of the **green fluorescent protein** (**GFP**). GFP was discovered in jellyfish and has become one of the most widely used 'tools' in biology, biochemistry, and biotechnology because its innate glow allows scientists to observe biochemical processes that are otherwise invisible. In the CHEM 313 lab, you have learned numerous methods to visualize and quantify proteins that are otherwise clear and indistinguishable from water, nucleic acids, carbohydrates, and lipids. The fact that GFP glows independently once its produced allows us to observe the process of protein translation in real-time.

In this activity, you will observe the structure of GFP using augmented reality to better understand the structural features of GFP that support green fluorescence.

Load the GFP structure on your device:

a. Download the "Augment" app from the Appstore or Play store
b. Load the app on your device
c. Press the [SCAN] button
d. Scan this QR code:

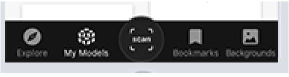

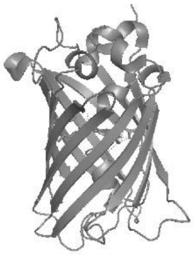

Open with your device's browser

e. Once the structure has been loaded, take a few minutes to play with the interface. In the main viewing mode, the GFP structure image will appear projected on a surface on your screen.
   - Use 1 finger to move the structure
   - Use 2 fingers to rotate the structure, and use a pinching motion to zoom in and out.

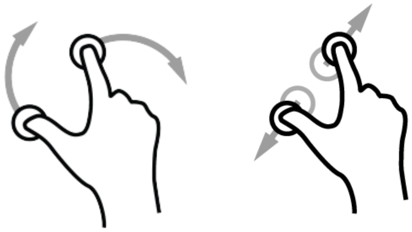

   - Press "3D view" to interact with your structure in more detail. In this mode, the GFP will be suspended in a black 3D space, other than on a surface.
     - In '3D view', you can make move and rotate the structure with 1 finger. Use a pinching motion to zoom in and out.

Green Fluorescent Protein – Augmented Reality Activity

1. The GFP that you see is a continuous polypeptide chain. Does this represent a primary, secondary, tertiary, or quaternary structure?

2. The structure of GFP is said to be a “barrel”. What type of secondary structure is making up the ‘wall’ of this barrel? Are they parallel or anti-parallel?

   a. Draw a cartoon of this type of secondary structure, noting locations of side chains, and the hydrogen bonding that hold the secondary structure together.

   b. Based on what you know about this secondary structure, where should the amino acid side chains be pointing, relative to the walls of the “barrel”?

While most proteins are transparent to the naked eye, GFP is unique in its ability to fluoresce. Fluorescence is the result of two phenomena, first is excitation followed by emission. GFP is capable of fluorescence after it is translated because three amino acids within the barrel react with each other to form a “Chromophore”, a unique molecule that can undergo excitation and emission at specific wavelengths of light.

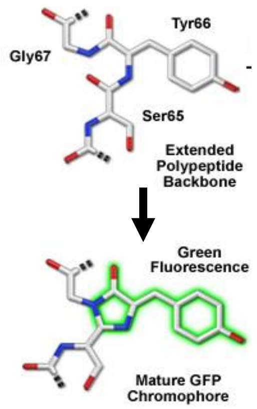

Rotate the structure so that you’re looking down through the barrel and identify the chromophore within your structure.

3. What are the full names of the three amino acids that make up the chromophore? Draw the structures of their side chains below.

Intermolecular forces are important for the structure and function of GFP. In addition to the hydrogen bonds holding the barrel together, hydrogen bonding to the chromophore are essential for GFP fluorescence.

Rotate and zoom into the barrel of your protein to find the dashed lines within the GFP barrel. The dashed lines the hydrogen bonds between the chromophore and other parts of the protein.

4. Identify 3 different hydrogen bonds that you observe in the structure. Name the amino acid of the chromophore that is participating in the hydrogen bond, note which atom is participating in the hydrogen bond, and note whether it is a donor or acceptor in the hydrogen bond.

    a.

    b.

    c.

5. What level of protein structure do these hydrogen bonds identified in question 4. represent? Primary, secondary, tertiary, or quaternary structure?

6. If GFP were to acquire a mutation to one of the amino acids above, what effect would this have on GFP fluorescence?

7. If GFP were to acquire a mutation to one of the amino acids that the chromophore pairs with, what effect would this have on GFP fluorescence?
