# Supplementary material for: The Genetic Code Kit: An Open-Source Cell-Free Platform for Biochemical and Biotechnology Education
Source: Front Bioeng Biotechnol. 2020 Aug 19;8:941. doi: 10.3389/fbioe.2020.00941 (PMC7466673; doi:10.3389/fbioe.2020.00941)
Supplement: Supplementary file 5 [file Data_Sheet_4.pdf]

For questions **1-4**: **match each process with the enzyme involved in catalysis** (A-D; each may be used once, more than once, or not at all)

A. Polymerase
B. Helicase
C. Ribosome
D. None of the above

1. Replication
2. Translation
3. Transcription
4. Peptide bond formation
5. Phosphodiester bond formation

6. Which of the following occurs during the synthesis of a new RNA strand?

A. the double-stranded DNA template is permanently changed into single-strands
B. a new phosphodiester linkage is formed between the 5' carbon atom of the DNA template and 3' carbon atom of the RNA being extended
C. a new phosphodiester linkage is formed between the 5' carbon atom of a ribonucleotide and the 3' carbon atom of a growing RNA strand
D. a new phosphodiester linkage is formed between the 5' carbon atom of a ribonucleotide and the 3' carbon atom of the DNA template
E. a new peptide bond is formed between the 5' carbon atom of a ribonucleotide and the 3' carbon atom of a growing RNA strand

7. Which of the following must occur on the ribosome?

A. Charging of a tRNA with an amino acid
B. Non-covalent interaction between mRNA and tRNA
C. Non-covalent interaction between mRNA and DNA
D. Formation of covalent bonds between nucleotides

For questions **8-11**, **match each process with the type of RNA involved in the given function** (A-D, each may be used once, more than once, or not at all).

A. messenger (mRNA)
B. transfer RNA (tRNA)
C. ribosomal RNA (rRNA)
D. all of the above

8. This type of RNA is translated into protein products
9. This type of RNA is required for the process of transcription
10. This type of RNA is covalently linked to the growing polypeptide chain during translation
11. This type of RNA is required for the process of translation

For questions **12**-**16**, use the following choices (each may only be used once)

A. at the AUG codon
B. at the UAG codon
C. at the terminator
D. at the promoter
E. at the ribosome-binding site/Shine-Dalgarno sequence

12. Where does **transcription** begin?
13. Where does **transcription** end?
14. Where does the **ribosome assemble**?
15. Where does **translation** begin?
16. Where does **translation** end?

Attitudinal questions:

	A	B	C	D
**17.** Transcription	*I have no idea what this term means.*	*I have heard this term before.*	*I have some familiarity with what this term means.*	*I know what this term means.*
**18.** Translation	*I have no idea what this term means.*	*I have heard this term before.*	*I have some familiarity with what this term means.*	*I know what this term means.*
**19.** Promoter	*I have no idea what this term means.*	*I have heard this term before.*	*I have some familiarity with what this term means.*	*I know what this term means.*
**20.** Terminator	*I have no idea what this term means.*	*I have heard this term before.*	*I have some familiarity with what this term means.*	*I know what this term means.*
**21.** RNA Polymerase	*I have no idea what this term means.*	*I have heard this term before.*	*I have some familiarity with what this term means*	*I know what this term means.*
**22.** Ribosome	*I have no idea what this term means.*	*I have heard this term before.*	*I have some familiarity with what this term means*	*I know what this term means.*
**23.** Cell-free protein synthesis	*I have no idea what this term means.*	*I have heard this term before.*	*I have some familiarity with what this term means*	*I know what this term means.*
**24.** Fluorescence	*I have no idea what this term means.*	*I have heard this term before.*	*I have some familiarity with what this term means.*	*I know what this term means.*

	A.	B	C	D	E
**25.** I am comfortable conducting experiments with enzymes	*Strongly Disagree*	*Disagree*	*Neutral*	*Agree*	*Strongly Agree*
**26.** I am comfortable conducting experiments with *in vitro* transcription	*Strongly Disagree*	*Disagree*	*Neutral*	*Agree*	*Strongly Agree*
**27.** I am comfortable conducting experiments with *in vitro* translation	*Strongly Disagree*	*Disagree*	*Neutral*	*Agree*	*Strongly Agree*
**28.** I am comfortable using micropipettes to work with small volumes (<100 uL)	*Strongly Disagree*	*Disagree*	*Neutral*	*Agree*	*Strongly Agree*
