# Supplementary material for: The Genetic Code Kit: An Open-Source Cell-Free Platform for Biochemical and Biotechnology Education
Source: Front Bioeng Biotechnol. 2020 Aug 19;8:941. doi: 10.3389/fbioe.2020.00941 (PMC7466673; doi:10.3389/fbioe.2020.00941)
Supplement: Supplementary file 6 [file Data_Sheet_5.pdf]

Supplementary Material

Supplementary Data

Supplementary Data Sheet 1: Genetic Code Kit Lab Manual

Listed in supplementary files.

Supplementary Data Sheet 2: Genetic Code Kit Student Worksheet

Listed in supplementary files.

Supplementary Data Sheet 3: Genetic Code Kit Augmented Reality Activity

Listed in supplementary files.

Supplementary Data Sheet 4: Pre-/Post- Questionnaire

Listed in supplementary files.

Supplementary Figures and Tables

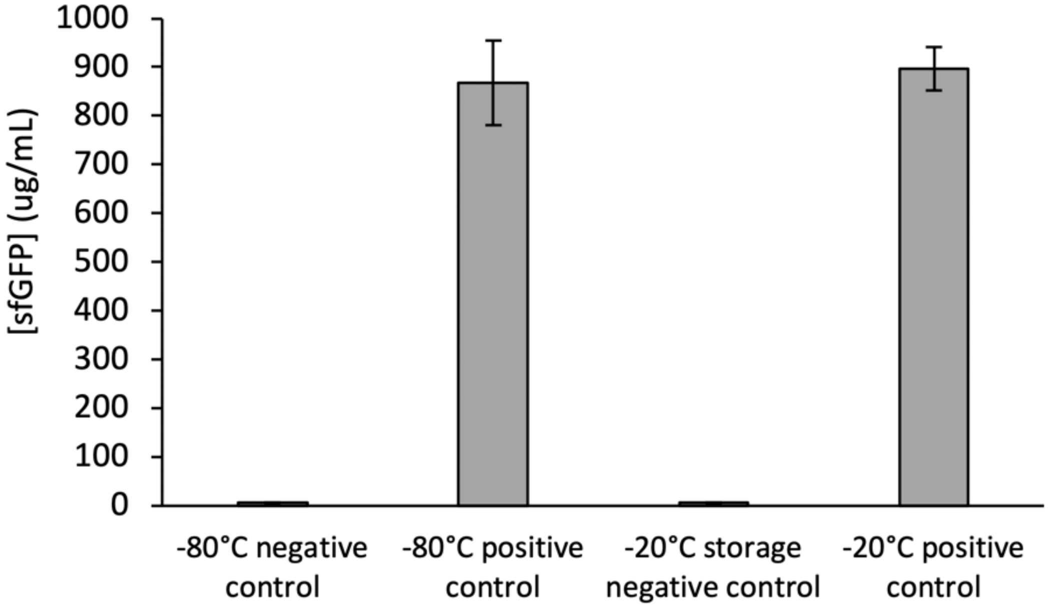

Supplementary Figure 1. CFPS productivity after storage of solutions A and B at -80 ºC and -20 ºC for 3 months. Aliquots of solutions A and B were stored in a -80 ºC research-grade freezer and a -20 ºC manual defrost (non-cycling) freezer, to mimic storage in a consumer-grade freezer. After 3 months, solutions were used to setup CFPS reactions with cell extract stored at -80 ºC. Negative control indicates that no DNA template was added.

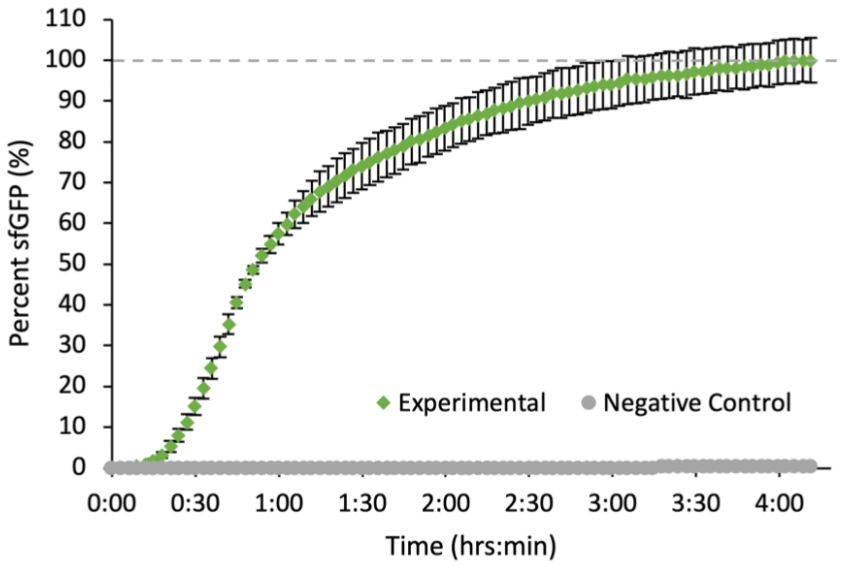

Supplementary Figure 2. Time-course analysis of a typical CFPS reaction producing sfGFP. Maximum protein production occurs between 3 and 4 hours, however a visible green color can be observed as early as 1-1.5 hours. Negative control indicates that no DNA template was added.

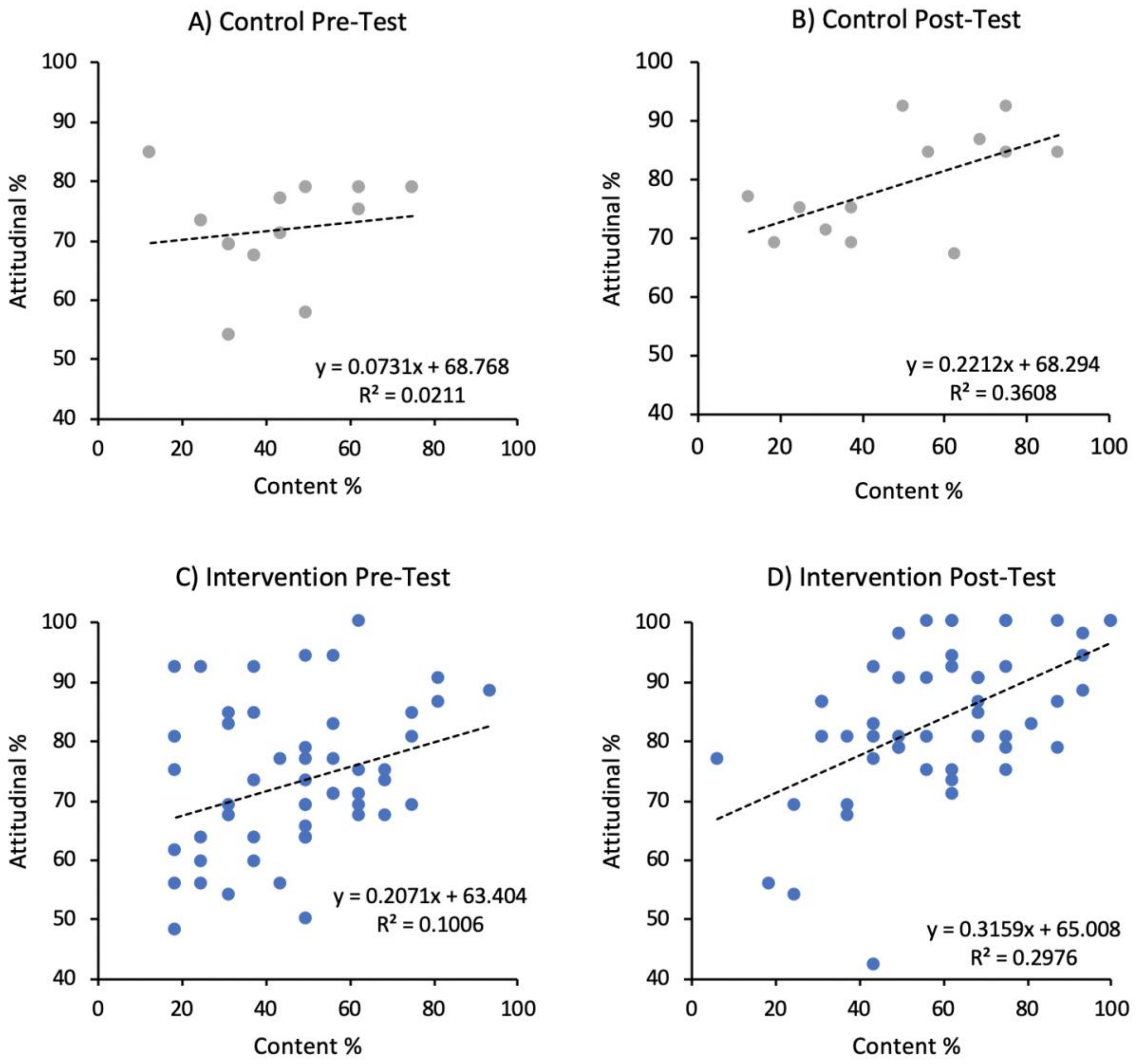

Supplementary Figure 3. Correlations between content-based scores and responses to attitudinal questions. Attitudinal values were obtained by converting multiple choice answers to numerical values (A=1, E=5) and determining each student's score out of the total possible points if the highest confidence was selected for each question. Each point represents one student, with control having 13 students, and intervention having 52 students. This is less than the number of students in the content analysis, as some did not complete the post-attitudinal section of the questionnaire.

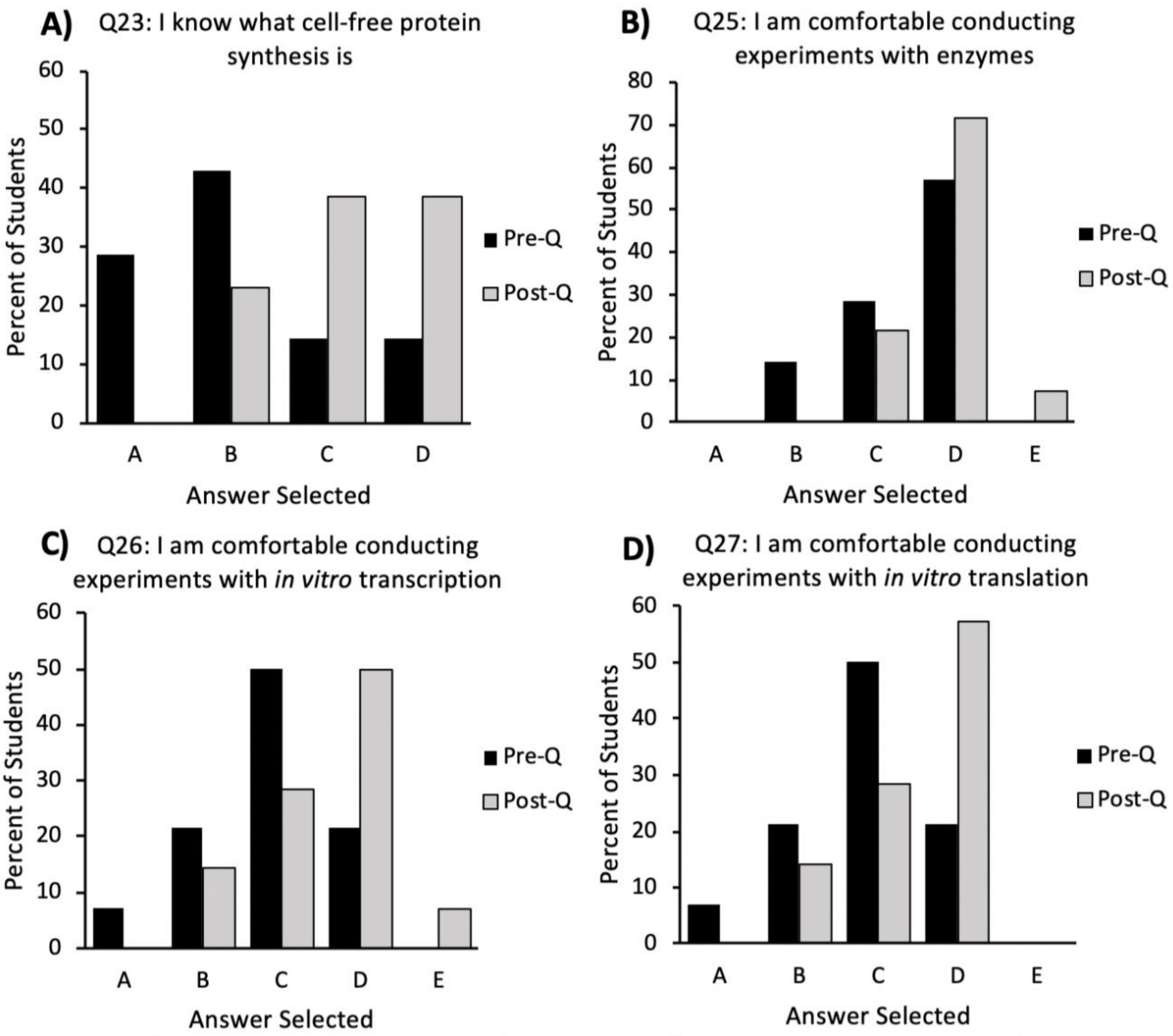

Supplementary Figure 4. Changes in control group student attitudes toward CFPS and conducting CFPS-based experiments. Answer choices for A) ranged from A – “I have no idea what this term means” to D – “I know what this term means”. Answer choices for panels B) – D) ranged from A – “Strongly disagree” to E – “Strongly agree.” Student answers were converted to a numerical value where A=1 and E=5, in order to calculate p-values using a one-sided paired t-test with a null hypothesis that pre- and post- scores would be equal. The control group contained 13 students. This is less than the number of students in the content analysis, as some did not complete the post-attitudinal section of the questionnaire. All possible answer categories can be found in Supplementary Data Sheet 2.

Supplementary Table 1: Anonymized Raw Data

Listed in supplementary files

Supplementary Table 2: Data Analysis Dashboard

The data analysis dashboard and corresponding readme file are included in the zip file for file-type compatibility.

In total, there are 69 students and 15 of them belong to the control group. As noted before, all students filled a 16 part questionnaire — the questions belong to three groups. Specifically, each student answered each question before the questionnaire (pre-questionnaire) and after the questionnaire (post-questionnaire). Then, for each question labelled i in {1,2,3,…,16}, there are four possibilities. Either a student incorrectly answered the question incorrectly during both pre- and post-questionnaire or a student answered the question correctly during the pre- and post- questionnaire. Since there was no improvement, we label these two possibilities as “No Change”. The third possibility implies that a student may have answered a question correctly during a pre-questionnaire and answered it incorrectly during the post-questionnaire — their outcome worsened. Aptly, we label this possibility as “Worse Off”. The last possibility concerns improvement and we label that possibility as “Improvement”.

Mathematically, whether there is Improvement, or whether a student is Worse Off or whether there is No Change is random. For a student i and for each question j in {1, 2,3,…,16}, we define a new variable X_{ij} that maps the possibilities into a numerical score:

$$X_{ij} = \begin{cases} 1 & Improvement \\ 0 & No\ Change \\ -1 & Worse\ Off \end{cases}$$

Last, we define an overall score for student i, X_i, where

$$X_i \equiv \sum_{j=1}^{j=16} X_{ij}$$

Keeping with the notation, we say that the student’s score improved overall if $X_i > 0$; the student is worse off if $X_i < 0$. If $X_i = 0$, then we say that there was no change. The data analysis dashboard shows the results for both the control and intervention group. Two observations are in order. First, the proportion of students who are worse off — percentage of students with $X_i < 0$, is less for the intervention group relative to the control group. Second, as a corollary, the proportion of students who improve — percentage of students with $X_i > 0$, is greater for the intervention group relative to the control group.

Supplementary Table 3:

This table shows the proportion of students who are either worse off or improved in the control group relative to the intervention group. We compare the results between the control and intervention groups statistically using the Fisher's Exact Test. Since the number of students in the control group is small relative to the intervention group, Fisher's Exact Test is a conservative test accounting for the small sample. The null hypothesis is that the proportion of students who are either worse off or improve is the same in the control group relative to the intervention group. We set the confidence threshold to 5%. That is, we reject the null if the p-value is lower than 5%.

Panel A: Differences between control and intervention group for Baseline questions			
	Proportion in the Control Group	Proportion in the Intervention Group	Statistically Significant
Worse off	33.33%	20.37%	No
Improved	33.33%	38.89%	No
Panel B: Differences between control and intervention group for TX/TL questions			
	Proportion in the Control Group	Proportion in the Intervention Group	Statistically Significant
Worse off	33.33%	20.37%	No
Improved	33.33%	66.67%	Yes
Panel C: Differences between control and intervention group for TX/TL questions without Q7 & 11			
	Proportion in the Control Group	Proportion in the Intervention Group	Statistically Significant
Worse off	46.67%	20.37%	No
Improved	40.00%	70.37%	Yes
Panel D: Differences between control and intervention group students who completed Ochem I for TX/TL questions without Q7 & Q11			

	Proportion in the Control Group	Proportion in the Intervention Group	Statistically Significant
Worse off	50.00%	21.74%	No
Improved	25.00%	69.57%	Yes
Panel E: Differences between control and intervention group students who completed Survey of Ochem for TX/TL questions without Q7 & Q11			
	Proportion in the Control Group	Proportion in the Intervention Group	Statistically Significant
Worse off	42.86%	21.88%	No
Improved	57.14%	68.75%	No
Panel F: Differences between control and intervention group students in the College of Agriculture, Food, and Environmental Science for TX/TL questions without Q7 & Q11			
	Proportion in the Control Group	Proportion in the Intervention Group	Statistically Significant
Worse off	33.33%	17.24%	No
Improved	66.67%	68.97%	No
Panel G: Differences between control and intervention group students in the College of Science and Mathematics, Engineering, and Liberal Arts for TX/TL questions without Q7 & Q11			
	Proportion in the Control Group	Proportion in the Intervention Group	Statistically Significant
Worse off	55.56%	24.00%	No
Improved	22.22%	72.00%	Yes
